# Validity of a Wheelchair Rugby Field Test to Simulate Physiological and Thermoregulatory Match Outcomes

**DOI:** 10.3390/sports10100144

**Published:** 2022-09-23

**Authors:** Fabian Grossmann, Joelle Leonie Flueck, Bart Roelands, Romain Meeusen, Claudio Perret

**Affiliations:** 1 Sports Medicine, Swiss Paraplegic Centre, 6207 Nottwil, Switzerland; 2 Human Physiology and Sports Physiotherapy Research Group, Vrije Universiteit Brussel, 1050 Brussels, Belgium

**Keywords:** exercise, performance, performance-assessment, Paralympic sport, body core temperature

## Abstract

The purpose of the study was to verify the criterion-validity (concurrent) of an existing and reliable, submaximal wheelchair Rugby (WCR) field test by examining the correlations of selected measures of physical performance between the field test and real games. Therefore, ten WCR athletes were observed during two WCR real games and during completing the field test two times. Total distance, mean and peak velocity, playing time, number of sprints, sprints per minute, mean and maximal heart rate, body core temperature (Tc), sweat rate, body weight loss, rate of perceived exertion and thermal sensation were measured. Values were correlated with the data observed by completing the field test two times separated by seven days. The results showed significant correlations between games and field tests for sweat rate (*r* = 0.740, *p* < 0.001), body weight loss (*r* = 0.732, *p* < 0.001) and the increase of Tc (*r* = 0.611, *p* = 0.009). All other correlations were not significant. For perceptual responses Bland–Altman analysis showed data within the limits of agreement. Descriptive statistics showed similarity for mean velocity and total distance between tests and games. In conclusion the study provides the first indications that the submaximal field test seems comparable with the game outcomes in terms of increase in Tc, covered distance, mean velocity and perceptual responses. Nevertheless, more research and additional validation are required.

## 1. Introduction

Laboratory- or field-based performance test protocols allow researchers to simulate sport specific performances or its physiological components in a more or less controlled scientific setting [1]. The major advantage of a field-based setting is that this allows testing under real-life conditions (e.g., sports hall with own sports-specific wheelchair), while it does not allow to provide the same level of standardization as laboratory tests [2]. In general, researchers have to consider three important points when selecting a protocol. First, the protocol must show a high reliability. Second, the chosen test is able to detect small changes in performance (sensitivity). Third, the protocol should mimic the real competition outcome as close as possible (validity) [1]. A protocol can be reliable without being valid but it cannot be valid without being reliable [3].

Most laboratory tests use an incremental or a continuous protocol and therefore, they do not have a high external validity for team sports with an intermittent character (alternation between high and low intensity activity) [4] such as wheelchair rugby (WCR) or wheelchair basketball [5,6]. In wheelchair team sports, e.g., wheelchair basketball or WCR several reliable field tests exist to assess maximal performance [6,7,8], which is very well summarized in the work of Goosey-Tolfrey and Leicht [5]. Gee, Lacroix and West [6] assessed agreement between peak physiological responses during game play and field-based 20 × 20 m intermittent sprint performance. They found moderate agreement for peak heart rate (bias: −2.22, 95% CI: −25.45 to 21.00) and good agreement for peak blood lactate, collected during the field test and game play (bias: −1.84, 95% CI: −6.86 to 3.18). Thus, these two parameters seem to be useful to assess changes in performance in response to interventions. Since they did not track the game with an indoor tracking system, which would have provided other parameters such as peak velocity or total distance, the validity of these parameters could not be assessed. Even though the beforementioned tests were able to replicate some peak physiological demands (e.g., peak heart rate, peak blood lactate concentration) of the sports [6], a valid field test which can mimic further performance parameters of a WCR game, such as mean heart rate, mean velocity, number of sprints, total covered distance and rate of perceived exertion, as well as thermal and thermoregulatory responses like sweat rate (SR) and increases of body core temperature (Tc), is missing. The assessment of validity would be necessary to assess whether, e.g., trainings or nutritional intervention or the uses of precooling techniques would have an influence on performance. Especially the testing of precooling techniques seems important because individuals with spinal cord injury (SCI) have limited thermoregulatory functions [9] and therefore an increased risk for heat-related injuries. Grossmann et al. [10] recently developed a reliable sub-maximal field test protocol for WCR with very small standard error of measurement and as well an excellent smallest real difference (for details please consider the article). Until now, it is unclear if this test protocol meets the above-mentioned criterion of validity. Therefore, the aim of this study was to verify the criterion-validity (concurrent) [1] of an existing submaximal WCR field test [10] by examining the correlations between the field test and selected measures of physical performance during real games. The authors hypothesized that the field test will be able to mimic physiological and thermoregulatory demands of a WCR game.

## 2. Materials and Methods

### 2.1. Participants

Ten national-level WCR players [age: 37.6, inter quartile range (IQR) 6.3 years, body mass: 75.5, IQR 23.9 kg] participated in this study (detailed characteristics are presented in Table 1). Nude body mass was assessed by weighing athletes on a specific scale for individuals with SCI (Paul Busch Waagenfabrik GmbH & Co., Hagen, Germany), level of lesion and American Spinal Injury Association Impairment Scale (AIS) were clinically determined during rehabilitation and confirmed during the classification process. The players were members of two different WCR teams, which were playing in the highest Swiss WCR league. Inclusion criteria were age between 18 and 60 years, male, spinal cord injury (SCI) with a valid classification for WCR. Exclusion criteria were the inability to swallow the core temperature pill and non-traumatic SCI (i.e., spina bifida, multiple sclerosis). All participants were informed about the experimental protocol, both orally and written and everyone provided their written, informed consent prior to data collection. The study was approved by the local ethical committee EKNZ (Basel, Switzerland) and registered under clinicaltrials.gov (NCT03815708). All procedures were conducted according to the Helsinki Declaration [11].

### 2.2. Study Design

**Figure 1:**
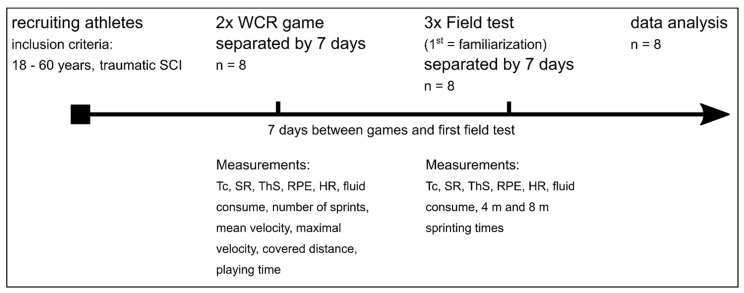
Study design; WCR = wheelchair rugby; n = number of participants; Tc = body core temperature; SR = sweat rate; ThS = perceived thermal sensation; RPE = rate of perceived exertion: HR = heart rate.

To assess data, participants had 5 appointments in total. They had to participate in two WCR games as well as to perform three times the field test. The first field test session was used as familiarization trial. Each study visit was separated at least 7 days (Figure 1).

### 2.3. Game-Assessment

Match-playing data were assessed during two competitive WCR games (Swiss League), played over four quarters of 8 min (effective time) on two separate days. Participants played in their own WCR-specific wheelchairs, which were weighed. Wheel diameter and camber angle were determined and type of tire and tire pressures were measured (Ralf Bohle GmbH, Schwalbe Airmax Pro, Reichshof, Germany) and noted individually. Games were played in an indoor sports complex with a hardwood flooring, typical for indoor wheelchair team court sports. The natural environmental conditions were 20.3 °C ± 0.6 °C with 49% ± 3% relative humidity. The games were played by all ten participants. Eight hours prior to the game a pill to analyze Tc (e-Celsius performance capsule, BodyCap, Hérouville-Saint-Clair, France) was ingested. For each player, the connection between the monitor (e-Celsius performance moniteur, BodyCap, Hérouville-Saint-Clair, France) and the pill was checked one hour before the game. Additionally, players were equipped with a heart rate monitor (Acentas heart rate monitoring belt, Acentas GmbH, Hoergertshausen, Germany). An indoor tracking system (Ubisense, Series 700 IP, Cambridge, UK) [14] was used to assess position data of four athletes which was later converted into distance travelled and speed parameters (mean speed, peak speed). Athletes performed an individual warm-up with a duration of 10 min at a subjective perceived exertion of 10 on a 6 to 20 Borg-scale [15]. Heart rate and Tc were collected during the warm-up. Immediately after the warm-up, athletes were weighed (PUA579-CS300, Mettler Toledo, Columbus, OH, USA) in their WCR chair (without the match jersey) and the drinking bottles were weighed (XS402S, Mettler Toledo, Columbus, OH, USA) as well. Directly before the game, participants were asked to rate their perceived thermal sensation (ThS) on a visual analogue scale [16] and the overall perceived exertion (RPE) on a Borg scale ranging from 6 to 20 [15]. Data collection of the performance parameters started with the beginning of the match clock and was only paused during a time-out, breaks between quarters and when a player was on the bench. During the game Tc was collected every 10 s. The mean of six values was used to calculate the one-minute-mean of Tc. Delta Tc was calculated by assessing the difference between the baseline and end of game Tc. For maximal Tc the highest one-minute-mean was used. Heart rate was measured with a remote monitoring system with a sample rate of one Hz (Acentas Herzfrequenz Monitoring Team System, Acentas GmbH, Hoergertshausen, Germany) and was paused analogue to the tracking system. Playing time for each participant was assessed by a commercially available stopwatch (DELTA, Sport-Thieme Germany, Grasleben, Germany). Water bottles were weighed throughout the game. Directly after the game athletes were toweled dry and were weighed in their WCB chair (without the match jersey). Fluid loss was calculated (pre-weight − post-weight + fluid consumption). Rating of ThS and RPE were assessed and Tc data were transferred from the pill to the monitor. Players were not allowed to use cooling techniques prior or during the game.

### 2.4. Field Test Assessment

Field test data were assessed during two sessions separated by seven days. The natural environmental conditions were 21.1 °C and 48% rh at session one, and 20.9 °C and 52% rh at session two and the tests were conducted in the same indoor sports complex. The test protocol was the same as developed by Grossmann and colleagues (2022). Athletes had to complete four sets of eight laps. Each lap represented 64 m, which had to be covered within one minute. A metronome was used for pacing purposes. Each lap consisted of one four-meter sprint, one eight-meter sprint and several stops, turns and wheeling parts at moderate intensity. Sprints had to be performed at maximal effort. Pre- and post-test procedures were replicated one-to-one from the games. In order to be sure that the athletes were familiar with the test, a familiarization trial was held seven days before the test session.

### 2.5. Statistics

For statistical analysis mean values of both tests and mean values of both games were used. Data were analyzed using the software R (R Foundation for Statistical Computing version 3.6.0; Vienna, Austria). After checking all measured parameters for normality using Shapiro Wilk’s tests, median and interquartile range (IQR) were calculated for all parameters. To check the criterion-related validity the games were used as the reference criterion and the Spearman rank correlation between the mean values of the two field test and the two games was calculated afterwards [1,17]. The meaningfulness of the size of the correlation coefficients were determined analogous to Mukaka [18]: *r* = 0.00 to 0.29 means negligible, 0.30 to 0.49 low, 0.50 to 0.69 moderate, 0.70 to 0.89 high and 0.9 to 1.0 very high relations. The Wilcoxon signed-rank test was used to assess the statistical difference between the mean values of both games and both field test sessions. The Bland–Altman method was used to examine the agreement between field tests and games. The mean difference between test and retest and SD of the difference and 95% limits of agreement [19]. Level of significance was set at a level of *p* < 0.05.

## 3. Results

**Figure 2:**
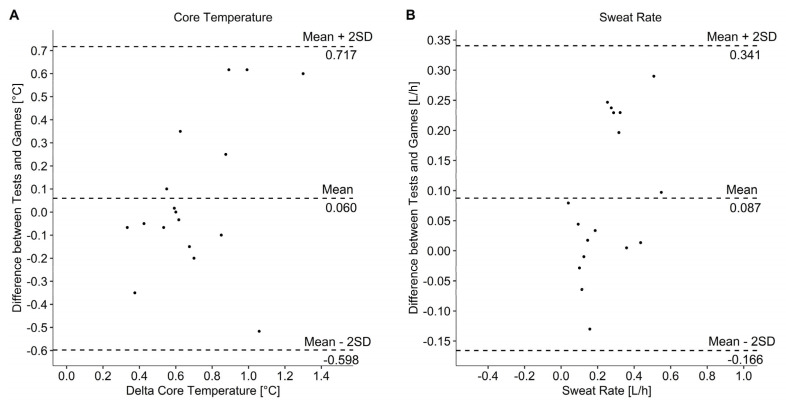
Bland–Altman plot for delta core temperature (**A**) and sweat rate (**B**). The middle, dashed line represents the mean difference between field tests and games. The other two dashed lines represent the limits of agreement.

**Figure 3:**
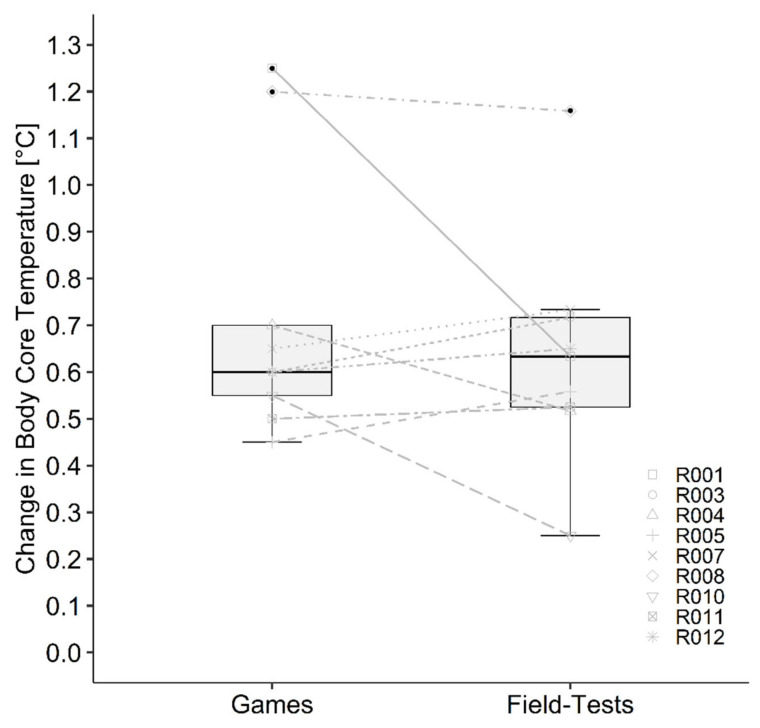
Change in body core temperature during the games and field tests for each participant. Boxplots demonstrate the median, interquartile range and distribution of the data. Lines between games and field tests demonstrate the individual difference in change of body core temperature between the games and field tests.

**Figure 4:**
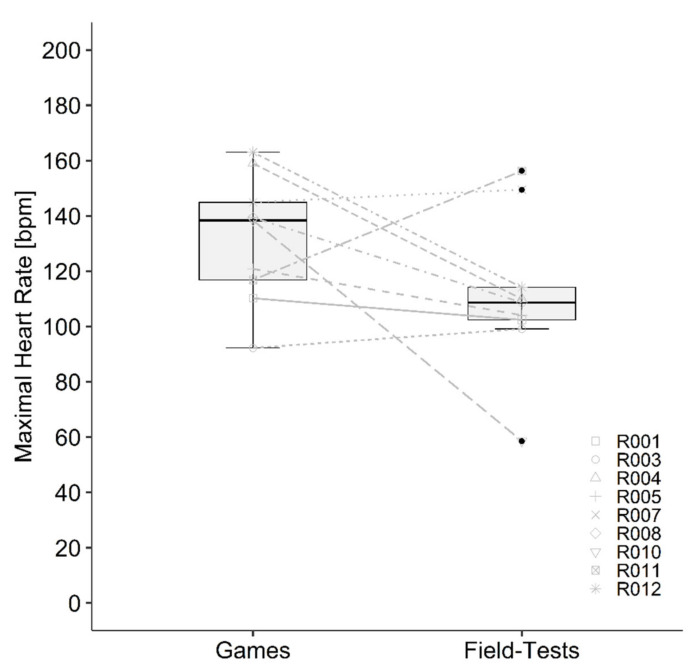
Mean heart rate during the games and field tests for each participant. Boxplots demonstrate the median, interquartile range and distribution of the data. Lines between games and field tests demonstrate the individual difference in mean heart rate between the games and field tests.

**Figure 5:**
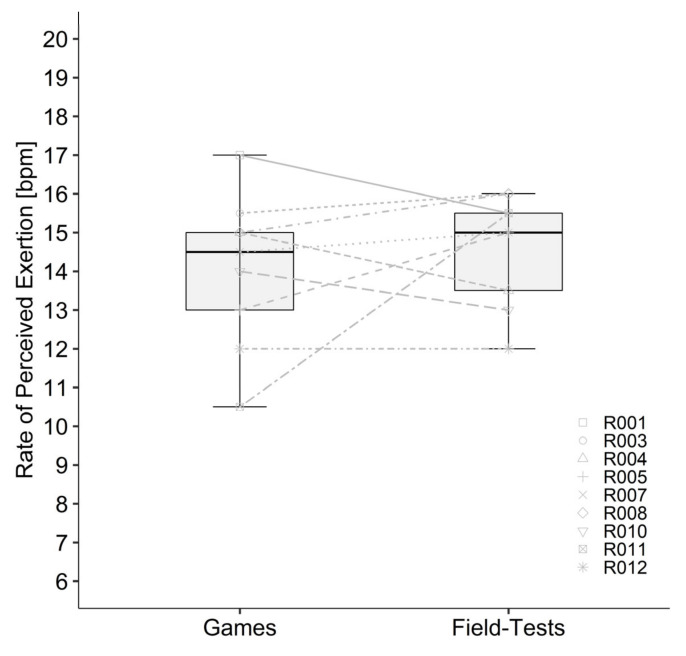
Rate of perceived exertion during the games and field tests for each participant. Boxplots demonstrate the median, interquartile range and distribution of the data. Lines between games and field tests demonstrate the individual difference in rate of perceived exertion between the games and field tests.

**Figure 6:**
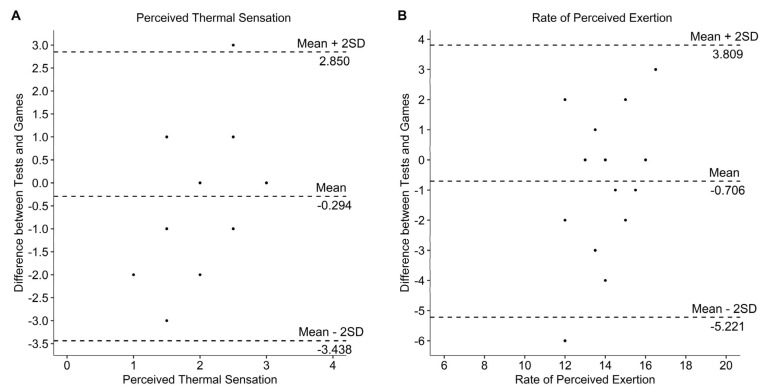
Bland–Altman plot for perceived thermal sensation (**A**) and rate of perceived exertion (**B**). The middle, dashed line represents the mean difference between field tests and games. The other two dashed lines represent the limits of agreement.

Data of the two field tests and games as well as the correlation between both games and both field tests are presented in Table 2 and Table 3. Statistics showed a significant correlation of SR (*r* = 0.740, *p* < 0.001) and body weight loss (*r* = 0.732, *p* < 0.001) and a significant moderate correlation for the increase of Tc (*r* = 0.611, *p* = 0.009). All other calculated correlations were not significant and between low and negligible. Bland–Altman plots showed agreement between field test and games (Figure 2). Descriptive statistics of tracking data (due to technical problems only four athletes with participation, in total 30 quarters) showed similarity for mean velocity and total distance. Number of sprints were considerably higher in the field test compared with the games. The mean values of the two games and the two field tests were significantly different for mean Tc (*p* = 0.005), max Tc (*p* = 0.011), SR (*p* = 0.014), fluid consumption (*p* < 0.001) and body weight loss (*p* < 0.001) and are presented in Table 3. Change in Tc (Figure 3), mean HR (Figure 4) RPE (Figure 5) and ThS were not different between the games and field tests. In perceptual responses Bland–Altman analysis demonstrated agreement between field test and games (Figure 6). Baseline values measured directly before the beginning of the warm-up did not significantly differ between the games and the field tests for any parameter except Tc. Tc was significantly lower before the field test warm-ups (37.0 vs. 37.4 °C, *p* = 0.003). 

## 4. Discussion

This study assessed the criterion-validity of thermoregulatory and physiological responses as well as performance characteristics of a WCR field test [10]. Furthermore, the field test was compared with two real games. The preliminary findings indicate that the field test seems to be able to replicate thermoregulatory responses like the increase in Tc and SR of WCR games. Descriptive statistics were comparable between the field test and the games concerning performance characteristics like mean velocity or total distance. The mean values of the two games and the two field tests were significantly different for mean Tc, max Tc, SR, fluid consumption and body weight loss and are presented in Table 3.

The approach to use a reliable, submaximal, sport-specific field test trying to simulate WCR games is new in this field. Since each WCR game can have its completely own character it was a challenging goal to check this reliable field test for the criterion-validity. Since the nature of the field test is to use fixed distance, mean velocity, number of sprints and playing time, these performance characteristics could only be compared descriptively and additionally with the current literature in this research field.

### 4.1. Performance Parameters

Total distance covered in the real games showed a high inter-individual variability (median: 2348 m; IQR: 2186 m), whereas each player covered 2048 m in the field test. Unfortunately, only four players have been tracked by the tracking system during the games and therefore, this distance possibly does not reflect the reality for all players. When comparing those values with data from the literature large differences were found: Sarro et al. [20] observed a total distance of 4540.1 ± 817.4 m, Griggs et al. [21] a total distance of 4842 ± 324 m. If we extrapolate mean distance of quarters found by Rhodes, Mason, Perrat, Smith, Malone and Goosey-Tolfrey [4], total distance was ~3700 m. This seems to be lower compared to the values found by Sarro, Misuta, Burkett, Malone and Barros [20], Griggs, Havenith, Price, Mason and Goosey-Tolfrey [21]. Since distance covered during short breaks was often included in the above-mentioned studies, total distance could be overrated as well. Therefore, Sarro, Misuta, Burkett, Malone and Barros [20] analyzed the distance covered only when the stop-clock was running. They found that only about 60% of the total distance is covered when the game is running. If this is true, total distance covered in the study mentioned above would be ~2700 m [20], which is similar to the distance in the present study. Additionally, classification seems also to have an influence on the covered distance. With regard to the values observed by Sarro, Misuta, Burkett, Malone and Barros [20] for athletes with a higher lesion level, the values (~2300 m) are even closer to the distance covered in our field test, where all athletes tracked showed a high lesion level as well.

When dividing the covered distance by the playing time, the similarity of the field tests to the games is given as well (field test: 64 m/min, games: 58.2 m/min). The assessed performance parameters during the game were comparable to the values found in the work by Rhodes, Mason, Perrat, Smith, Malone and Goosey-Tolfrey [4]. Since the four players tracked during both games all had a classification between 0 and 1.5 points, the assessed values can be compared with those of group I and II in the study by Rhodes, Mason, Perrat, Smith, Malone and Goosey-Tolfrey [4], in which the relative distance for full played quarters ranged between 59.9 and 69.7 m/min. Mean velocities of the field test were slightly higher compared to the games (1.08 m/s vs. 1.03 m/s). Rhodes, Mason, Perrat, Smith, Malone and Goosey-Tolfrey [4] showed a similar mean velocity (1.08 ± 0.12 m/s). Sarro, Misuta, Burkett, Malone and Barros [20] (1.13 ± 0.20 m/s) and Griggs, Havenith, Price, Mason and Goosey-Tolfrey [21] (1.13 ± 0.11 m/s) found slightly higher values, whereas it seems worth mentioning that the majority of the athletes included here were with a high classification [20]. Rhodes, Mason, Perrat, Smith, Malone and Goosey-Tolfrey [4] defined sprints as high intensity events. The number of high intensity events ranged between 44 and 52 per game. In the present games the median number of sprints was 20 with an IQR from 4 to 46 (some players played only short parts of the games). These numbers show that the 64 sprints performed in the field test were too high and not even players who played the whole game reached this number. Comparing time in movement between the field test and the games similar but inverted patterns were shown. Whereas the field test duration was fixed at 32 min, the time the players were in movement seemed to be higher in the real games (median: 39.5 IQR: 28.7 min, range: 22:17–66:13 min). Additionally, Gavel et al. [22] observed a large variability in overall movement time (27:43 ± 9:40 min (range: 12:10–43:10)) during a WCR game. Since WCR players also remain active when the game clock was stopped the duration was longer than the effective playing time. In terms of overall intensity, it is speculated, that the higher number of sprints compensates the lower covered distance.

### 4.2. Physiological Parameters

Medians of mean and peak heart rate did not correlate between the field tests and the two games even if the median values were similar (field test: 103 bpm, games: 104 bpm). Compared with the literature the median of both games (median: 104, IQR: 28 bpm) was similar to the value (100 ± 20 bpm) Griggs, Havenith, Price, Mason and Goosey-Tolfrey [21] had observed. Since a real WCR game has significantly more stoppages than the field tests, it is interesting that the test reached similar values. One reason can be that the noticeable higher peak heart rate values reached during the game (129 vs. 110 bpm) have compensated the lower values during the stoppages. The time between the sprints in the field tests seems to be sufficient to return to pre-sprint level, which confirms the submaximal character of the test. As observed by the authors during the games, it often occurs that one sprint is directly followed by the other, providing insufficient time in between to reach pre-sprint heart rate values. Thus, the athletes reached higher peak heart rates. In the games the participants reached similar peak heart rate values (range: 88–168 bpm) as seen in previous work (range: 92–154 bpm) [23]. Additionally, these peak heart rate values fit in the values (range: 112–171 bpm) seen in the maximal 20 × 20 m field test of Gee, Lacroix and West [6]. Important to mention is that the level and the completeness of lesion is a determinant of the peak heart rate [24]. In individuals with a TP the heart rate regulation is limited due to the autonomic dysfunction. Therefore, values between different studies with different athletes are only comparable to a limited extent [25,26].

### 4.3. Thermal and Thermoregulatory Responses

Significant correlations were found for thermal and thermoregulatory responses (SR, body weight loss and for the increase in Tc). Even if mean Tc and maximal Tc did not coincide, the field test was able to simulate a similar increase of Tc (median 0.6, IQR: 0.2 °C) as measured in the games (median 0.6, IQR: 0.3 °C). Interestingly our observed data did not match with Tc increases during WCR games (1.6 ± 0.4 °C) seen in previous research [21]. It can be speculated that this considerably higher change in Tc can be a reason of a more intense game. Athletes covered noticeable more distance (4842 ± 324 m) with a higher mean velocity (1.13 ± 0.11 m/s), thus generating a greater amount of heat stress [21], which resulted in a higher increase of Tc. Additionally, they played ~71 min on average, which is substantially longer as in the presented study. Figure 2 shows that participants R001, R004 and R010 had a larger increase in Tc during the game. One reason for this obvious difference compared to the other players is the significantly longer playing time for these three participants. The significantly higher mean and max Tc observed in the games were presumably also a consequence of the prominent difference in playing time. This speculation can be verified by the study of Gavel, Lacroix (18), which showed a relation between the increase in Tc and movement time per quarter. Additionally. the different limitation in thermoregulation due to SCI [9] should be taken into account when comparing individual data.

Even when the SR was significantly higher in the games (0.39 L/h) compared to the field test (0.17 L/h), a significant high correlation was shown. Bland–Altman plot shows data within limits of agreement. Since SR is very variable from day to day and depends on different external factors [27] a significant different may be present quickly. Therefore, it can be speculated that SR is as well replicated by the field test in a good way. Black et al. [28] found a similar SR (0.17 ± 0.21 L/h) during WCR trainings with a game situation (25 min warm-up and training-game 5 vs. 5). This supports the findings that the field test can simulate the thermal response of a WCR game.

Perceptual values (i.e., RPE and ThS) showed no significant correlations between the tests and the games, nevertheless the range for peak RPE was quite similar (games: median 14, range 11–16, field test: median 15, range 11–16) and the statistics showed no significant differences between games and field tests. The agreement shown in Bland–Altman plot (Figure 6) can additionally confirm the similarity between field tests and games. Griggs, Havenith, Price, Mason and Goosey-Tolfrey [21] showed an RPE of 16 during a real game. Consequently, it can be speculated that RPE is a useful parameter to assess perceived exertion.

For ThS the data are similar. Even though there was no correlation between the games and the field test, the individual numbers show the contrary. In the field test the median ThS was 2 with an IQR of 1, for the games the median was as well 2 and the IQR 2. Concerning this point, we think that these findings can be an issue of the use of such scales. Most people do not perceive the categories of the scale as equidistant and therefore the range of sensations regarding a specific point on the scale varies largely [29]. Thus, any statistical analysis has to be taken with caution.

### 4.4. Limitations

The recruitment of an appropriate number of participants is difficult since athletes with SCI represent a small group in the athletic population. Therefore, a careful balance between not overrating significant findings and devaluating insignificant results has to be kept in mind. This problem is accentuated in the present work, since performance data with the indoor tacking system were only generated out of four athletes who played the two games. WCR rugby is played by both male and female athletes, but in this study, only male athletes were included. Therefore, the results of this study cannot be transferred to all athletes. Each WCR game has its own character, therefore a comparison of two different studies which analyzed different games, with different participants, with different performance levels, is difficult. In the present study, only WCR players with a traumatic SCI were included. Therefore, it is unclear if the field test would be reliable/valid for players with another type of disability (e.g., amputation, cerebral palsy). As a consequence, more studies with a higher number of participants with similar inclusions and exclusions criteria are needed.

### 4.5. Practical Applications

In WCR researchers and coaches can use this field test to assess whether the effect of different training or nutritional interventions and as well the application of different cooling techniques have an impact on performance or thermoregulatory interventions. The results would help to further develop WCR and as well each athlete individually. Additionally, it is recommended to generate more data to further assess the validity of the field test.

## 5. Conclusions

In conclusion, the field test is comparable with WCR games in terms of the increase of Tc, total covered distance and mean velocity. Other physiological values as mean HR or peak HR did not show a significant correlation, although they seemed to be comparable and agreed quite well with the already existing literature. For perceptual values (ThS and RPE) the data showed no correlation between the field tests and the games, nevertheless Bland–Altman analysis showed most values within the limit of agreement. The current study provides the first indications that the current, reliable submaximal field test can be valid.

## Figures and Tables

**Table 1:** Participants’ characteristics.

ID	Age [Years]	Body Mass [kg]	Lesion Level	AIS	Classification
1	36.2	91.9	C6	A	0.5
2	49.0	78.0	C5	A	0.5
3	37.9	92.6	C5	C	1.5
4	28.7	63.7	C5	C	0.5
5	42.6	67.0	C6	B	1.5
6	45.4	64.6	C7	A	2
7	42.2	77.3	C6	D	2
8	37.3	89.8	C8	D	3
9	25.3	73.6	C7	D	2.5
10	32.2	68.0	C7	D	2
Median, IQR	37.6, 6.3	75.5, 23.9		-	-

IQR = interquartile range; C = cervical lesion; AIS = American Spinal Injury Association (ASIA) Impairment Scale [12]; Classification referred to International Wheelchair Basketball Federation (IWBF) [13].

**Table 2:** Field test vs. games “game characteristics”, n = 4.

	Test 1		Test 2		Test Combined		Game 1		Game 2		Games Combined		Correlation between Tests and Games	
Measured Parameter	MED	IQR	MED	IQR	MED	IQR	MED	IQR	MED	IQR	MED	IQR	*r*	*p*-Value
Total distance [m]	2048	-	2048	-	2048	-	2693	2331	2283	1874	2348	2186	-	-
rel. distance [m/min]	64	-	64	-	64	-	60.4	3.1	52.0	2.9	58.2	8.2	-	-
V_mean_ [m/s]	1.08	-	1.08	-	1.08	-	1.01	0.06	0.90	0.04	1.03	1.75	-	-
Number of sprints	64	-	64	-	64	-	26	33	17	28	20	35	-	-
rel. sprint [sprint/min]	2	-	2	-	2	-	0.5	0.3	0.3	0.4	0.5	0.5	-	-

MED = median; IQR = interquartile range; *p* = *p*-value; *r* = correlation coefficient; rel. = relative; V_mean_ = mean velocity; m = meter; m/min = meter per minute; m/s = meter per second. Columns with a grey background show the average data of both tests or games.

**Table 3:** Field test vs. games, “physiological and thermoregulatory parameters”, n = 10.

	Test 1		Test 2		Tests Combined		Game 1		Game 2		Games Combined		Correlation between Tests and Games		Game vs. Field Test
Measured Parameter	MED	IQR	MED	IQR	MED	IQR	MED	IQR	MED	IQR	MED	IQR	*r*	*p*-Value	*p*-Value
Playing time [min]	32	-	32	-	32	-	42.3	30.0	36.7	20.6	39.5	28.7	-	-	-
HR_mean_ [bpm]	98.4	16.9	103.24	13.30	103	14	118	22	118	22	104	28	0.328	0.197	0.459
HR_max_ [bpm]	106	19	110	22	110	14	121	32	140	32	129	32	0.416	0.097	0.080
RPE [Borg]	15	2	16	1	15	2	15	1	15	1	14	2	0.100	0.400	0.286
ThS	2	0.3	2.5	1	2	1	2	1.8	0	1	2	2	0.353	0.408	0.372
Tc_mean_ [°C]	37.3	0.3	37.4	0.4	37.4	0.4	37.9	0.6	37.9	0.54	37.9	0.6	0.232	0.367	0.005 ^†^
Tc_max_ [°C]	37.7	0.2	37.9	0.3	37.8	0.3	38.25	1.1	38.3	0.5	38.3	1.0	0.256	0.320	0.011 ^†^
Tc_Delta_ [°C]	0.6	0.4	0.7	0.2	0.6	0.2	0.65	0.35	0.6	0.3	0.6	0.3	0.611	0.009 *	0.831
Sweat rate [L/h]	0.15	0.05	0.19	0.13	0.17	0.09	0.4	0.22	0.16	0.31	0.39	0.31	0.740	<0.001 *	0.015 ^†^
Fluid consume [L]	0	0	0	0	0	0	0.39	0.28	0.26	0.36	0.29	0.32	0.123	0.636	<0.001 ^†^
Weight loss [kg]	0.12	0.04	0.18	0.13	0.14	0.14	0.57	0.33	0.23	0.41	0.53	0.56	0.732	<0.001 *	<0.001 ^†^

MED = median; IQR = interquartile range; *p* = *p*-value; *r* = correlation coefficient; rel. = relative; V_mean_ = mean velocity; HR_mean_ = mean heart rate; HR_max_ = maximal heart rate; RPE = rate of perceived exertion; ThS = thermal sensation; Tc_mean_ = mean body core temperature; Tc_max_ = maximal body core temperature; Tc_Delta_ = increase in body core temperature; * significant correlation at a level of *p* < 0.05, † significant difference between games and field tests at a level of *p* < 0.05. Columns with a grey background show the average data of both tests or games.

## Data Availability

The datasets used and analyzed during the current study are available from the corresponding author on reasonable request.
